# Assessment of Vault Particles in Cancer Cell Line‐Derived Extracellular Vesicle Preparations

**DOI:** 10.1002/jev2.70142

**Published:** 2025-08-06

**Authors:** Xinming Liu, Zubair Ahmed Nizamudeen, Christopher J. Hill, Christopher Parmenter, Kenton P. Arkill, Daniel W. Lambert, Stuart Hunt

**Affiliations:** ^1^ School of Clinical Dentistry The University of Sheffield Sheffield UK; ^2^ Translational Medical Sciences, The University of Nottingham Biodiscovery Institute The University of Nottingham Nottingham UK; ^3^ Cryo‐Electron Microscopy Facility The University of Sheffield Sheffield UK; ^4^ Nanoscale and Microscale Research Centre The University of Nottingham Nottingham UK

**Keywords:** extracellular vesicles, major vault protein, vault particle, vault RNA

## Abstract

Extracellular vesicles (EVs) may contain a variety of molecular cargo, including proteins and nucleic acids. Vault particle components have been repeatedly reported in the literature as EV cargo. Here, we demonstrated that vault RNA (vtRNA) and major vault protein (MVP) were highly abundant in EV pellets enriched by differential centrifugation (DC) by qPCR and western blotting, respectively. RNase and proteinase treatment of DC‐derived pellets demonstrated that most vtRNA and MVP were not enclosed and protected within an EV membrane. Vault‐like particles were visualised in 100k DC pellets by cryo‐transmission electron microscopy. EVs were enriched by size exclusion chromatography, and western blotting of individual fractions showed co‐elution of EV markers and vault particle proteins. Immunocapture of EVs post‐ultracentrifugation (100k DC pellet) showed co‐purification of MVP, whereas EVs isolated by direct immunocapture from conditioned medium were MVP‐negative. The current study highlights the importance of determining the topology of putative EV‐associated components to determine if they are EV cargo or contaminants that have been co‐purified.

## 1 Introduction

Since the discovery that extracellular vesicles (EVs) can transfer functional RNA between donor and recipient cells (Valadi et al. 2007; Kosaka et al. 2010), there has been a massive increase in studies profiling EV RNA cargo (Batagov and Kurochkin 2013; Crescitelli et al. 2013; Huang et al. 2013; Li et al. 2013; Cheng et al. 2014). These findings have indicated that EVs contain a large variety of RNA species, including ribosomal RNA (rRNA), transfer RNA (tRNA), microRNA (miRNA), messenger RNA (mRNA), long and short non‐coding RNA (ncRNA), Y RNA, and vault RNA (vtRNA).

VtRNA are ∼100 nt small non‐coding RNA with a stem‐loop secondary structure (Van Zon et al. 2003). Three vtRNA paralogues are transcribed from the *VTRNA1* locus (vtRNA1‐1, vtRNA1‐2 and vtRNA1‐3) and one from the *VTRNA2* locus (vtRNA2‐1). In addition, two vtRNA pseudogenes (*VTRNA2‐2P* and *VTRNA3‐1P*) are annotated on the human genome assembly hg38 (Büscher et al. 2020). vtRNAs account for approximately 5% of the mass of the vault particle—the largest known ribonucleoprotein complex in eukaryotic cells, localising mainly in the cytoplasm (Kedersha and Rome 1986). Apart from the nucleic acid components, these 13 MDa subcellular organelles primarily consist of three vault proteins: major vault protein (MVP), telomerase protein component 1 (TEP1) and poly (ADP‐ribose) polymerase 4 (PARP4/vPARP). MVP accounts for over 70% of the particle mass, with the outer shell of the vault containing 78 MVP copies (Tanaka and Tsukihara 2012). Although their function has not been fully elucidated, vault particles and individual vault components have been associated with multiple cellular activities, including cytoskeleton transport, multi‐drug resistance, certain signalling pathway regulation and immunity (Li et al. 1999; Mossink et al. 2003; Berger et al. 2009; Gopinath et al. 2010).

Mining of the ExoCarta database revealed that MVP and vtRNA have been repeatedly reported as EV cargo or to be associated with EVs, with some studies linking them with potential intracellular trafficking and gene regulatory functions (Herlevsen et al. 2007; Nolte‐’t Hoen et al. 2012; Lässer et al. 2017). However, additional experimental work is required to assess if they are *bona fide* EV cargo. In 2014, the International Society for Extracellular Vesicles (ISEV) published a set of guidelines for EV research, which was then reviewed and updated in 2018 and 2023 (Lötvall et al. 2014; Théry et al. 2018; Welsh et al. 2024). These guidelines provided advice on using biochemical approaches to further assess the topological association of putative EV cargo to provide more convincing evidence (Théry et al. 2018; Welsh et al. 2024).

In 2019, Jeppesen et al. provided evidence that MVP and vtRNAs are released from cells in an exosome‐independent manner, and therefore should not be considered as exosome cargo (Jeppesen et al. 2019). The heterogeneous nature of EVs (released from the same cell and different cell types) raises the question: Are vault particles a common contaminant of EV preparations? If so, how can we achieve EV isolation that is free of vaults and other similar‐sized particles?

To address these questions, we compared three commonly used isolation methods (differential centrifugation, size exclusion chromatography, and Dynabead immunocapture) for enriching EVs from oral cancer cell line conditioned medium. We assessed the presence of EV and vault components, followed by biochemical approaches to confirm their topology/association with EVs. In the case of differential centrifugation‐derived EVs, pellets collected from three increasing centrifugal speeds were assessed individually, aiming to profile the presence of vault components in pellets commonly used to enrich distinct EV subtypes. We also aimed to develop a high‐purity EV isolation strategy, free from non‐EV‐related vault contamination. Taken together, this study explores the suitability of commonly used isolation methods in conducting EV cargo research and provides insight into vault‐free EV purification.

## 2 Materials and Methods

### 2.1 Cell Culture

Oral squamous cell carcinoma (OSCC) cell lines, H357 and SCC4, were routinely cultured in keratinocyte growth medium (KGM) (Allen‐Hoffmann and Rheinwald 1984) supplemented with 10% (v/v) fetal bovine serum (FBS) (Sigma). The final concentration of KGM components was: 67% (v/v) low glucose Dulbecco's Modified Eagle's Medium (DMEM), 23% (v/v) Nutrient Mixture F‐12 Ham, 100 IU·mL^−1^ penicillin and 100 µg·mL^−1^ streptomycin, 2.5 µg·mL^−1^ amphotericin B solution, 2 mM L‐glutamine, 1.8 × 10^−4^ M adenine, 0.5 µg·mL^−1^ hydrocortisone, 5 µg·mL^−1^ human insulin, and 10 ng·mL^−1^ human Epidermal growth factor (hEGF). Cells were maintained in a humidified cell culture incubator at 37°C with 5% CO_2_.

### 2.2 Differential Centrifugation

Differential centrifugation methodology was adapted from the protocol described by Théry et al. (Théry et al. 2006). Briefly, 2 million cells were seeded in T175 flasks and allowed to adhere. After 24 h, the growth medium was discarded, sub‐confluent monolayers were washed in PBS, and medium was replaced with fresh KGM supplemented with 10% (v/v) ultra‐filtered FBS (UF‐FBS) to deplete bovine EVs as described elsewhere (Kornilov et al. 2018). After 72 h, a cell confluence of approximately 80% was reached, and the conditioned medium was collected for differential centrifugation. Cell debris was first removed by centrifugation at 300 × *g* for 10 min. The supernatant was then taken to the next centrifugation step at 2000 × *g* for 10 min, followed by a wash with PBS and centrifugation at the same speed for another 10 min to generate a 2k pellet. Next, the supernatant was centrifuged at 10,000 × *g* for 30 min to produce a 10k pellet, followed by washing with PBS and pelleting for 30 min at the same speed. Finally, the supernatant was centrifuged at 100,000 × *g* for 1 h to generate a 100k pellet, followed by a PBS wash and centrifugation for 1 h at the same speed. Initial rounds of centrifugation were performed using an Avanti J‐26 XP centrifuge with a JA‐12 fixed‐angle rotor (Beckman Coulter), before ultracentrifugation using an Optima L‐90K centrifuge with a Type 45 Ti fixed‐angle rotor (Beckman Coulter). All centrifuge steps were performed at 4°C.

### 2.3 Size Exclusion Chromatography

After 72 h, conditioned medium was collected and centrifuged at 300 × *g* for 10 min. The supernatant was concentrated to 0.5 mL using a Vivaspin‐20 spin column (100 kDa MWCO) (28932363, Cytiva) by centrifuging at 6000 × *g* for ∼45 min. Concentrated conditioned medium was fractionated by size exclusion chromatography (SEC) by application to Sepharose CL‐2B resin (17014001, Cytiva) (14 mL Sepharose slurry added per column to give a 10 mL bed volume), stacked in a disposable Econo‐Pac column (7321010, Bio‐Rad). The column was eluted with PBS, and 0.5 mL fractions were collected. Fractions were stored at −20°C, ready for further analysis.

### 2.4 Dynabead Immunocapture

Dynabeads conjugated with mouse anti‐human CD63 (10606D, Invitrogen), CD9 (10614D, Invitrogen), and CD81 antibodies (10616D, Invitrogen) were mixed at a 100 µL:40 µL:40 µL ratio, respectively. The volumes used relate to the manufacturer's instructions for each set of Dynabeads. The Dynabead mixture was used to capture EVs positive for these three tetraspanin markers, according to the manufacturer's protocol. Mouse IgG isotype control antibody (sc‐2025, Santa Cruz Biotechnology) was conjugated with M‐450 Epoxy Dynabeads (14011, Invitrogen) according to the manufacturer's protocol. Tetraspanin and mouse IgG (mIgG) beads were washed in PBS with 0.1% (m/v) bovine serum albumin (BSA) (Sigma) prior to use. For further purification of EVs isolated by ultracentrifugation, 100k pellets were resuspended in 1 mL PBS, combined with either tetraspanin or mIgG beads (final concentration 1.14 × 10^7^ beads·mL^−1^) and incubated on an orbital shaker at 4°C overnight. Alternatively, conditioned medium was centrifuged at 300 × *g* for 10 min. Five millilitres of supernatant were concentrated to 1 mL using a Vivaspin‐20 spin column (100 kDa MWCO) and incubated with either tetraspanin or mIgG beads. After incubation, samples were placed on a magnetic separator to pull out tetraspanin‐positive EVs. The supernatant was carefully removed, which contained EVs negative for the three tetraspanins and other particles (i.e., the unbound fraction). The supernatant was ultracentrifuged at 100,000 × *g* in an Optima TLX benchtop ultracentrifuge with a TLA‐100.4 fixed‐angle rotor (Beckman Coulter) for 1 h to pellet unbound particles. Where necessary, the volume of sample contained in thick‐wall polycarbonate ultracentrifuge tubes (362305, Beckman Coulter) was corrected to the nominal 3 mL volume by addition of PBS before ultracentrifugation. The pelleting of unbound particles was done for all Dynabead immunocapture experiments. EVs captured by the magnetic Dynabeads were first washed with PBS with 0.1% (m/v) BSA, then twice with PBS. During each wash step, the samples were taken off the magnetic separator, and the beads were gently washed by pipetting. The samples were placed back onto the magnetic separator for 1 min before the wash buffer was removed, and the process was repeated for three washes total, followed by EV lysis for downstream western blot analysis.

### 2.5 Nanoparticle Tracking Analysis

Nanoparticle tracking analysis (NTA) was performed using a ZetaView PMX 110 instrument (Particle Metrix GmbH), which was calibrated with polystyrene particles of known size. Three millilitres of conditioned medium or diluted EV preparations were injected into the sample cell, followed by automated acquisition and analysis by the instrument software (ZetaView 8.04.02, Particle Metrix GmbH). DC pellets resuspended in PBS, individual SEC fractions, and combined SEC fractions were analysed using two distinct settings to detect small and large particles. Settings for small particles were: Sensitivity 85; Shutter 70; MinBright 25 pixels; MaxArea 500 pixels; MinArea 20 pixels; Framerate 30 frames per second; Tracelength 15; Video quality medium; Positions 11; 3 cycles. Settings for large particles were: Sensitivity 65; Shutter 90; MinBright 15 pixels; MaxArea 3000 pixels; MinArea 25 pixels; Framerate 3.75 frames per second; Tracelength 15; Video quality highest; Positions 11; 1 cycle. Particle concentrations were taken from the software‐generated report. Particle size and count data were taken from.txt files and used to calculate size profiles.

### 2.6 ExoView Analysis

EVs in conditioned medium were characterised using the ExoView R100 imaging platform (NanoView Biosciences) coupled with ExoView tetraspanin chips (NanoView Biosciences) that bind EVs positive for CD9, CD63, and CD81, with mouse IgG used as a negative control. The conditioned medium was first centrifuged at 300 × *g* for 10 min. The supernatant was then diluted (1/2–1/5) in a proprietary incubation solution and loaded onto an antibody‐coated chip. The chip was incubated at room temperature overnight, followed by several wash steps and incubation with fluorescent secondary antibodies. Finally, the chip was analysed by the ExoView R100 reader, and images were captured and analysed by the corresponding acquisition software ExoScan v0.998 (NanoView Biosciences).

### 2.7 Transmission Electron Microscopy

Prior to resin‐embedded TEM imaging, EV‐Dynabead complexes were fixed in fresh 2.5% glutaraldehyde in 0.1 M phosphate buffer (pH 7.4) overnight at 4°C, subsequently washed in 0.1 M phosphate buffer twice at 15 min intervals at 4°C before being post‐fixed in 2% aq. osmium tetroxide for 2 h at room temperature, then washed again in buffer as above. Samples were dehydrated through a graded series of ethanol in water, cleared in propylene oxide, and embedded in araldite resin for transmission electron microscopy. Ultrathin sections, approximately 70–90 nm thick, were cut on a Reichert Ultracut E ultramicrotome with a diamond knife and stained for 25 min with 3% aq. uranyl acetate, washed in water, followed by staining with Reynold's lead citrate for 5 min. For negative staining of EVs in suspension, samples were absorbed on discharged carbon‐coated copper grids for 5 min. Excess liquid was drained prior to staining with 1% phosphotungstic acid (pH 7.2) for 1 min. The grids were then washed twice in distilled water for 1 min, followed by imaging. Samples were imaged using a Tecnai T12 Spirit transmission electron microscope (FEI) at an accelerating voltage of 80 kV and images recorded with a Gatan Orius 1000B digital camera using Gatan digital micrograph software.

### 2.8 Cryogenic TEM

Sample preparation for cryo‐TEM was adapted from an existing protocol (Nizamudeen et al. 2018, Nizamudeen et al. 2021). Carbon film copper TEM grids were used (EM resolutions, Sheffield, UK). Samples were left to adsorb onto the grids (5 µL/grid) for 2 min, excess solution was removed using a filter, and samples were frozen using a Gatan CP3 plunge freezing unit (Ametek, Leicester, UK), blotted for 1 s, and frozen in liquid ethane. Cryo‐TEM was carried out using a Tecnai Biotwin‐12 at an accelerating voltage of 100 kV.

### 2.9 Western Blotting

EV pellets and EV‐Dynabead complexes were solubilised with RIPA buffer (20‐188, Millipore) supplemented with the cOmplete EDTA‐free protease inhibitor cocktail (04693159001, Roche) and 0.1% (v/v) Pierce universal nuclease (88700, Thermo Fisher Scientific). Lysates were centrifuged at 13,000 × *g* for 5 min, and the supernatant was kept on ice for immediate use or stored at ‐20°C. For experiments where an equal amount of total protein was loaded, protein concentrations were measured with the Pierce BCA protein assay kit coupled with the bovine serum albumin standards (23225, Thermo Fisher Scientific) according to the manufacturer's protocol. Samples were mixed with 5× loading buffer (EC‐887, National Diagnostics) and heated at 95°C for 5 min prior to the sample loading and separation by SDS‐PAGE on a 10% or 12% polyacrylamide gel. Proteins were then transferred using a Trans‐Blot Turbo Transfer System and the Trans‐Blot Turbo Mini Nitrocellulose Transfer Packs (1704158, Bio‐Rad), which were blocked with 5% (w/v) skimmed milk for 1 h at room temperature. Membranes were probed with the following antibodies (all purchased from Abcam unless otherwise stated), with dilution factor and final concentration provided: MVP [EPR13227(B)] (ab175239) 1:2000 (0.46 µg·mL^−1^), TEP1 (ab64189) 1:4000 (0.25 µg·mL^−1^), PARP4 [EPR8230] (ab133745) 1:1000 (2.73 µg·mL^−1^), CD9 [EPR23105‐121] (ab92726) 1:1000 (0.53 µg·mL^−1^), CD63 [EPR5702] (ab134045) 1:1000 (0.36 µg·mL^−1^), CD81 [EPR4244] (ab109201) 1:1000 (0.23 µg·mL^−1^), TSG101 [51/TSG101] (612697, BD Biosciences) 1:500 (0.5 µg·mL^−1^), GM130 [EP892Y] (ab52649) 1:1000 (0.12 µg·mL^−1^), anti‐rabbit IgG (7074, Cell Signaling Technology) 1:3000 (0.03 µg·mL^−1^), anti‐mouse IgG (7076, Cell Signaling Technology) 1:3000 (0.06 µg·mL^−1^). For chemiluminescence detection, the SuperSignal West Pico PLUS (34580, Thermo Fisher Scientific) and WESTAR Supernova HRP detection substrate (XLS3‐0100, Cyanagen) were used for high‐ and low‐abundance proteins, respectively. Blots were scanned on a C‐DiGit Blot Scanner (Li‐Cor) or otherwise exposed to a CL‐XPosure film (Thermo Fisher Scientific) and then developed and fixed on a Compact X4 developer (Xograph).

### 2.10 Proteinase K Protection Assay

Differential centrifugation pellets resuspended in PBS were divided into four aliquots of equal volume and treated with: (A) PBS only, (B) Proteinase K (Qiagen) diluted with PBS to 20 µg·mL^−1^ final concentration, (C) Triton X‐100 (Sigma) diluted with PBS to 0.1% (v/v) final concentration, and (D) Proteinase K (20 µg·mL^−1^ final concentration) and Triton X‐100 (0.1% (v/v) final concentration). All samples were then incubated at 37°C for 30 min before phenylmethanesulfonyl fluoride (PMSF, 5 mM final concentration, Sigma) was added and incubated for 10 min at room temperature to terminate proteinase digestion. Treated samples were analysed by western blotting.

### 2.11 RNA Extraction and Quantification

RNA extraction for small RNA sequencing was carried out using the miRCURY RNA isolation kit (300110, Exiqon). RNA was extracted according to the manufacturer's instructions. Due to unexpected discontinuity of the product, RNA extraction for other experiments was performed using the Monarch Total RNA Miniprep Kit (T2010S, New England Biolabs) according to the provided protocol.

### 2.12 Small RNA Sequencing

RNA sequencing was performed by the Edinburgh Clinical Research Facility (Edinburgh, UK) using the Ion Proton Platform (Thermo Fisher Scientific). Quality control checks and quantification were performed using an Agilent 2100 Electrophoresis Bioanalyzer instrument with the Agilent RNA 6000 Pico kit (Agilent Technologies), and a Qubit 2.0 fluorometer with the Qubit RNA HS Assay kit (Thermo Fisher Scientific). Using the Ion Total RNA‐Seq kit v2 with an optimised protocol for low amounts of short RNA cargos (<200 nt), the RNA was hybridised prior to cDNA reverse transcription and purification. cDNA was amplified with Ion Torrent adapters before the products were quantified with the Qubit 2.0 fluorometer and the dsDNA HS Assay kit, while the library size distributions were obtained on an Agilent Bioanalyzer with the DNA HS kit. Equal molar quantities of libraries were combined for template preparation before sequencing on an Ion Proton instrument using a P1 v3 chip. In addition to the automatically produced BAM files (by the instrument software), microRNA reads were examined using a small RNA analysis plugin v5.0.3.0, by which the reads were aligned to mature miRNAs. Any unmapped sequences were aligned to the whole genome and counted as other RNA molecules.

### 2.13 Quantitative Real‐Time PCR

Ten nanograms total RNA was reverse transcribed using a High‐Capacity cDNA Reverse Transcription Kit (4368814, Applied Biosystems) with random primers according to the manufacturer's protocol. Based on our small RNA sequencing data, three miRNAs with stable abundance across all samples (miR‐23a‐3p, miR‐30d‐5p, and miR‐31‐5p) were chosen to be used as internal controls. For miRNA, the TaqMan MicroRNA Reverse Transcription Kit (4366596, Applied Biosystems) was used, coupled with TaqMan MicroRNA Assay 5× RT primers (miR‐23a‐3p 000399, miR‐30d‐5p 000420, and miR‐31‐5p 002279) to reverse transcribe 10 ng of total RNA. To quantify RNA abundance, 2× qPCRBIO Probe Blue Mix (PB20.25‐01, PCR Biosystems) was combined with 20× TaqMan primers (vtRNA 1‐1 Hs03676993_s1, vtRNA 1–2 Hs06632430_gH, and vtRNA 1–3 Hs04330458_s1), nuclease‐free water, and cDNA (an equivalent of 250 pg RNA) for each reaction. For miRNA‐qPCR, miRNA‐cDNA template (an equivalent of 333 pg RNA) was used with 20× TaqMan primers specific for the above three miRNAs. qPCR was performed on a Rotor‐Gene Q real‐time PCR cycler (Qiagen), and a two‐step run was programmed: 10 min at 95°C for initial denaturation, followed by 40 cycles of 15 s at 95°C and 60 s at 60°C, in which the green channel was acquired during the second step. A threshold of 0.04 was set to obtain Ct values across all experiments. Raw Ct values were then analysed using the delta‐delta Ct method to generate the relative fold change of the transcript abundance compared to the selected control (Livak and Schmittgen 2001).

### 2.14 RNase A Protection Assay

Differential centrifugation pellets resuspended in PBS were divided into five aliquots of equal volume. Proteinase K (20 µg·mL^−1^ final concentration, Qiagen) was first added to the relevant samples, followed by incubation at 37°C for 30 min. 5 mM PMSF was added to all samples to halt proteinase activity by incubation at room temperature for 10 min. Triton X‐100 (0.1% (v/v) final concentration) and RNase A (20 µg·mL^−1^ final concentration, Invitrogen) were added to the appropriate samples followed by incubation at 37°C for a further 30 min, before RNaseOUT Recombinant Ribonuclease Inhibitor (Thermo Fisher Scientific) was added to all samples at a final concentration of 8 U·µL^−1^ with an incubation at room temperature for 5 min. Where reagents were added to some samples, an equal volume of PBS was added to the rest. RNA was then extracted from all samples as described above.

### 2.15 Nano Flowcytometry

A dual laser (488/640 nm) NanoAnalyzer U30 instrument (NanoFCM) was used for simultaneous quantification of side scatter and fluorescence of individual particles. Gravity‐fed HPLC‐grade water served as the sheath fluid, sampling pressure was 1 kPa and measurements were taken over 60 s. Particle concentrations were determined by comparison to standard 250 nm silica nanoparticles of known concentration (Quality control beads, NanoFCM). Particle sizes were determined by comparison to a mixture of nanospheres of four different diameters (S16M‐Exo, NanoFCM) by generating a standard curve based on the intensity of side‐scattered light of the four different silica particle populations of 68, 91, 113, and 155 nm in diameter. Prior to measuring samples, a blank measurement for PBS buffer was recorded to allow subtraction of background particle concentration. The concentration of unlabelled particles in DC‐enriched and SEC‐enriched EVs was measured. Particles were diluted to ∼2 × 10^10^ particles mL^−1^ in PBS. One microliter ExoBrite 515/540 True EV Membrane Stain (diluted 1:50 in PBS to produce a 10× solution) was added to 9 µL sample or 9 µL of PBS (serving as a control for non‐specific fluorescence), before incubation for 30 min at room temperature in the dark. Stained particles were diluted to ∼2 × 10^8^ particles mL^−1^ in PBS. Non‐specific fluorescence in the ExoBrite + PBS control was subtracted from the sample data.

### 2.16 Data Analysis and Statistical Analysis

Statistical analysis was performed (Prism 8, GraphPad), where data were presented as the mean ± standard deviation (SD). Data plotting was performed using Prism 8 (v. 4.2.2) and RStudio (v. 2023.06.0, Posit Software). Multiple *t*‐tests with Holm–Sidak test correction were used to confirm statistical significance where applicable (*p* < 0.05).

## 3 Results

### 3.1 Vault Particles Contaminate Differential Centrifugation EV Preparations

For all EV enrichment experiments, cell lines were incubated in medium supplemented with UF‐FBS, produced by the ultrafiltration method of Kornilov et al. (2018), which successfully depleted all detectable particles from the UF‐FBS (Figure S1). We first enriched EVs from H357 and SCC4 cell conditioned medium by differential centrifugation (DC), a method that has been used by over 80% of EV researchers (Gardiner et al. 2016). The protocol was adapted from that described by Théry et al. in 2006 (Théry et al. 2006). Conditioned medium was sequentially centrifuged at 2000 × *g*, 10,000 × *g*, and 100,000 × *g* to generate 2k, 10k, and 100k pellets, respectively.

**FIGURE 1:**
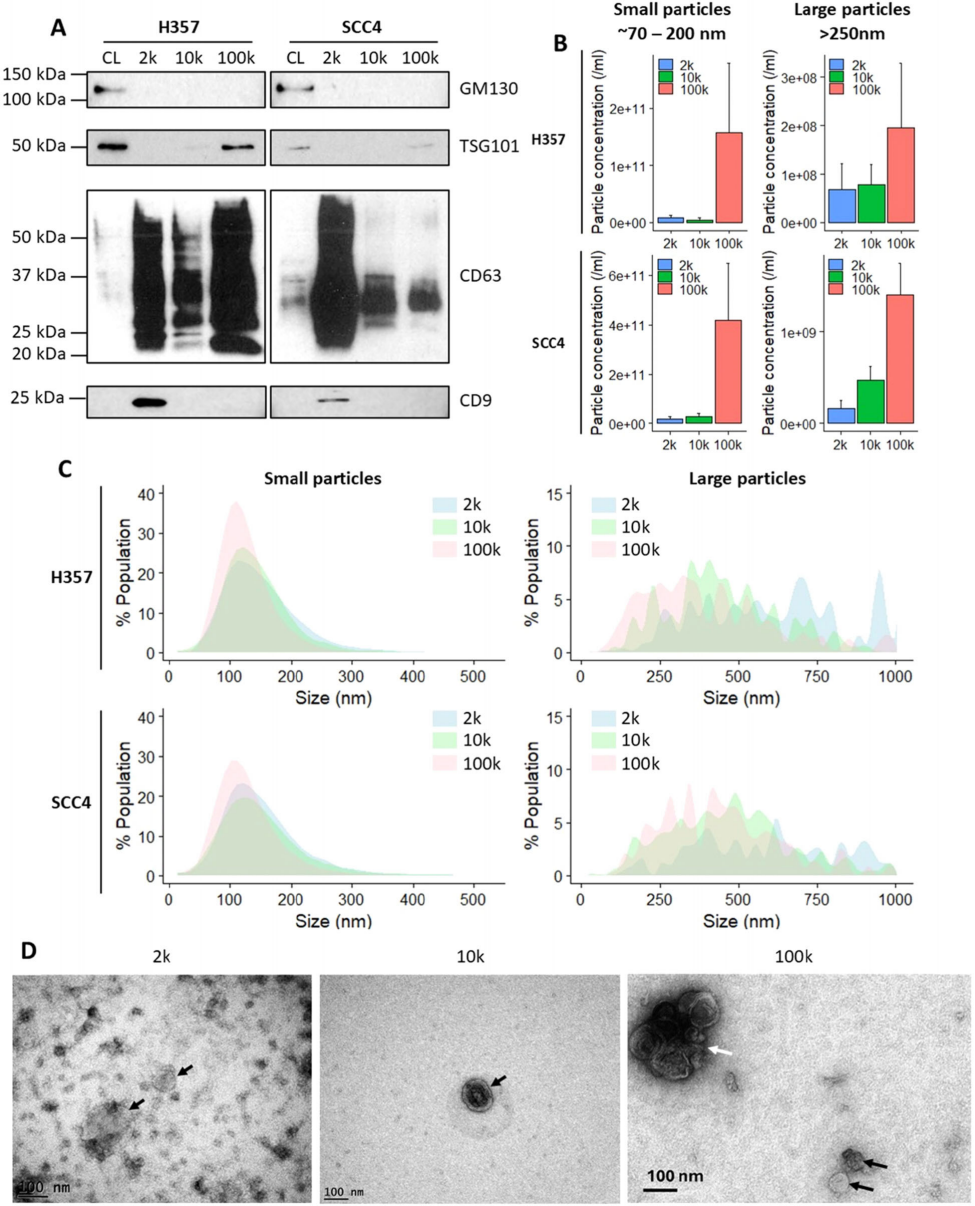
Differential centrifugation‐derived pellets contain EVs. (A) Western blots of H357 and SCC4 whole cell lysates (CL), 2000 × *g* (2k), 10,000 × *g* (10k), and 100,000 × *g* (100k) differential centrifugation pellets. Equal quantities (2 µg) of total protein were separated by SDS‐PAGE. Common EV markers (CD9, CD63, TSG101) and EV‐negative marker GM130 were probed. Blots are representative of three independent repeats. (B) ZetaView NTA showing particle numbers per mL of 2k, 10k, and 100k pellets from H357 and SCC4 cell lines. Small particles and large particles were measured using the corresponding settings on the instrument according to the manufacturer's instructions. Data shown are mean ± SD, *n* = 3. (C) ZetaView NTA showing the size distribution profiles of 2k, 10k, and 100k pellets from H357 and SCC4 cells using settings focusing on small and large EVs on the instrument. Data are the mean of three independent experiments. (D) Negative stain TEM analysis of 2k, 10k and 100k pellets from SCC4 cell line. Black arrows indicate individual EVs, and the white arrow indicates an aggregate of EVs. Scale bars 100 nm.

DC‐derived pellets were characterised according to the Minimal Information for Studies of Extracellular Vesicles 2023 (MISEV2023) guidelines (Welsh et al. 2024). Western blotting confirmed all DC‐pellets were enriched with the common EV marker CD63, 2k and 100k pellets were also positive for CD9 and TSG101, respectively, whilst GM130 (an intracellular Golgi apparatus protein) was only detected in the whole cell lysate (Figure 1A). Nanoparticle tracking analysis (NTA) was used to elucidate particle concentration (Figure 1B) and size distribution (Figure 1C) of small and large particles in DC‐derived pellets. Two different sets of NTA measurement parameters were used to quantify small (∼70–200 nm) and large (>250 nm) particles in each sample. Small particles were approximately 10‐fold enriched in 100k pellets compared to 2k and 10k pellets. Small particles in 100k pellets had a narrower size range, with a peak diameter ∼100 nm, whilst those in 2k and 10k pellets showed broader curves that included particles of larger diameter. As expected, by analysing the same samples using an NTA setting that focused on larger particles, we were able to detect broad particle size profiles, ranging from 50 to 1000 nm. Although more large particles were detected in 100k pellets than in 2k and 10k, these particles were mostly smaller than 500 nm. Whereas large particles in 2k and 10k pellets were enriched with those larger than 300 nm. Large particles in all pellets displayed multiple size peaks, in contrast to the single peaks observed in small particle analysis.

This could indicate the heterogeneous nature of larger extracellular particles, but could also result from the aggregation of smaller particles. By utilising two different NTA acquisition settings, we were able to gain less biased information regarding the size range of particles present in each pellet.

Negatively stained transmission electron microscopy (TEM) confirmed the presence of EVs in the DC pellets. The 2k pellet contained numerous negatively stained structures of varying size, whilst the 10k pellet contained EVs that displayed artefactual cup‐shaped morphology but were sparse across the TEM grid. The 100k pellet contained numerous EVs with the artefactual cup‐shaped morphology. Some individual EVs were present as well as in compact aggregates (Figure 1D). Larger electron micrographs are provided in Figure S2.

**FIGURE 2:**
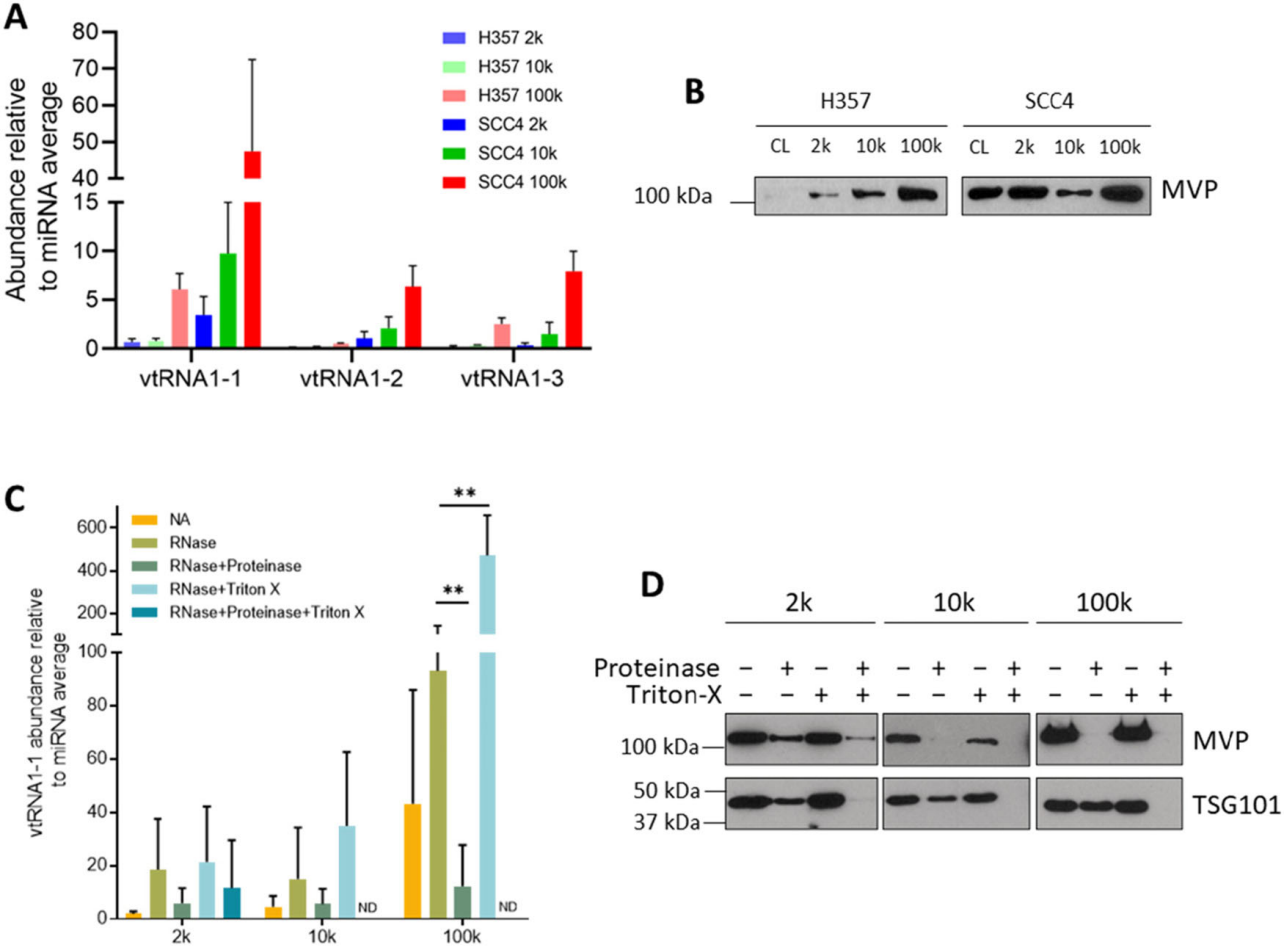
Vault components are not protected by an EV membrane. (A) qPCR analysis of vtRNA abundance in 2k,10k, and 100k DC pellets derived from H357 and SCC4 cells. VtRNA abundance is reported relative to three miRNAs (miR‐23a‐3p, miR‐30d‐5p, and miR‐31‐5p) that were chosen as endogenous controls based on small RNA sequencing data. Data are means ± SD, *n* = 3. (B) Western blotting of MVP abundance in cell lysates and DC pellets derived from H357 and SCC4 cells. Equal amounts of proteins were separated by SDS‐PAGE. Blots are representative of three independent experiments. (C) RNase protection assay of SCC4 DC pellets followed by qPCR to determine vtRNA1‐1 abundance. Data are means ± SD, *n* = 3 (ND = not determined due to insufficient RNA for qPCR analysis). Statistical significance was assessed by multiple t tests corrected with the Holm–Sidak method, **p < 0.01. (D) Proteinase protection assay of SCC4 DC pellets coupled with western blotting detecting major vault protein and the luminal EV marker, TSG101, upon proteinase and membrane‐permeabilising treatments. Blots are representative of three biological repeats.

Following confirmation that DC pellets contained EVs, we next characterised the abundance of vtRNA paralogues in 2k, 10k and 100k pellets by qPCR (Figure 2A). VtRNA1‐1 abundance was highest, followed by vtRNA1‐2 and vtRNA1‐3, with all paralogues enriched in 100k pellets (Figure 2A). This mirrored the abundance of vtRNA seen in a preliminary small RNA sequencing experiment of 10k and 100k pellets (Figure S3A and Table S1), which revealed two vtRNA paralogues in the top 20 most abundant RNA species in H357 and SCC4‐derived 100k pellets (Figure S3A and Figure S3B). We also detected MVP, the predominant protein component of the vault particle (Kedersha et al. 1990), in DC pellet lysates by western blotting (Figure 2B).

Therefore, we assessed whether the vault components present in DC pellets were bona fide EV cargo by biochemical assays. From this point, all experiments were carried out using the SCC4 cell line due to the higher concentration of extracellular particles released (Figure 1B), hence higher protein and RNA yields for downstream experiments. We first utilised an RNase protection assay coupled with qPCR to determine if vtRNA present in DC pellets was protected by an EV membrane (Figure 2C and Figure S3C). vtRNA abundance was reported relative to the average abundance of three miRNAs (miR‐23a‐3p, miR‐30d‐5p, and miR‐31‐5p) that showed abundant reads, which were consistent between DC pellets derived from the same cell line as determined by small RNA sequencing (Table S2). The data from treatment of 100k pellets was the most straightforward to interpret, most likely due to the enrichment of vtRNA in these samples (Figure 2A). RNase A treatment alone of 100k pellets was not sufficient to degrade vtRNA1‐1. However, pre‐treatment of 100k pellets with proteinase K, followed by RNase, resulted in a significant decrease in vtRNA1‐1 abundance (Figure 2C). Treatment of 100k pellets with Triton X‐100 detergent and RNase caused a significant increase in relative vtRNA1‐1 abundance, which was due to the selective degradation of the control miRNAs that were protected by an EV membrane (Figure S3D). Incubation of 100k pellets with proteinase K, Triton X‐100 and RNase A resulted in insufficient RNA remaining in most samples for qPCR analysis. Similar results were also observed for vtRNA1‐2 and vtRNA1‐3 (Figure S3C). Interrogation of raw qPCR data revealed a consistent ∼2 Ct value increase (i.e., decreased abundance) for all vtRNA paralogues in 100k pellets after proteinase K and RNase treatment compared to untreated samples. Whereas the Ct values for the control miRNA remained constant in 100k pellets after proteinase K and RNase treatment compared to untreated samples (Figure S3D). Therefore, the decrease in vtRNA abundance is specifically due to a decrease in vtRNA template and not a normalisation artefact due to increased control miRNA abundance. Taken together, these data suggest that the majority of extracellular vtRNA in 100k pellets are protected by protein‐shelled structures, rather than an EV membrane.

Similarly, we tested if the main vault structural component, MVP, was protected by an EV membrane. Proteinase treatment of 10k and 100k pellets in the absence of detergent revealed that MVP was completely digested and not protected by an EV membrane (Figure 2D). MVP in 2k pellets was not completely digested in any condition tested, which may be due to the large pellets produced (Figure 2D). In contrast, TSG101, a core component of the ESCRT‐I complex, a commonly accepted intraluminal EV marker (Katzmann et al. 2001; Théry et al. 2018), was only digested by proteinase in the presence of the membrane permeabilising detergent Triton X‐100 (Figure 2D).

**FIGURE 3:**
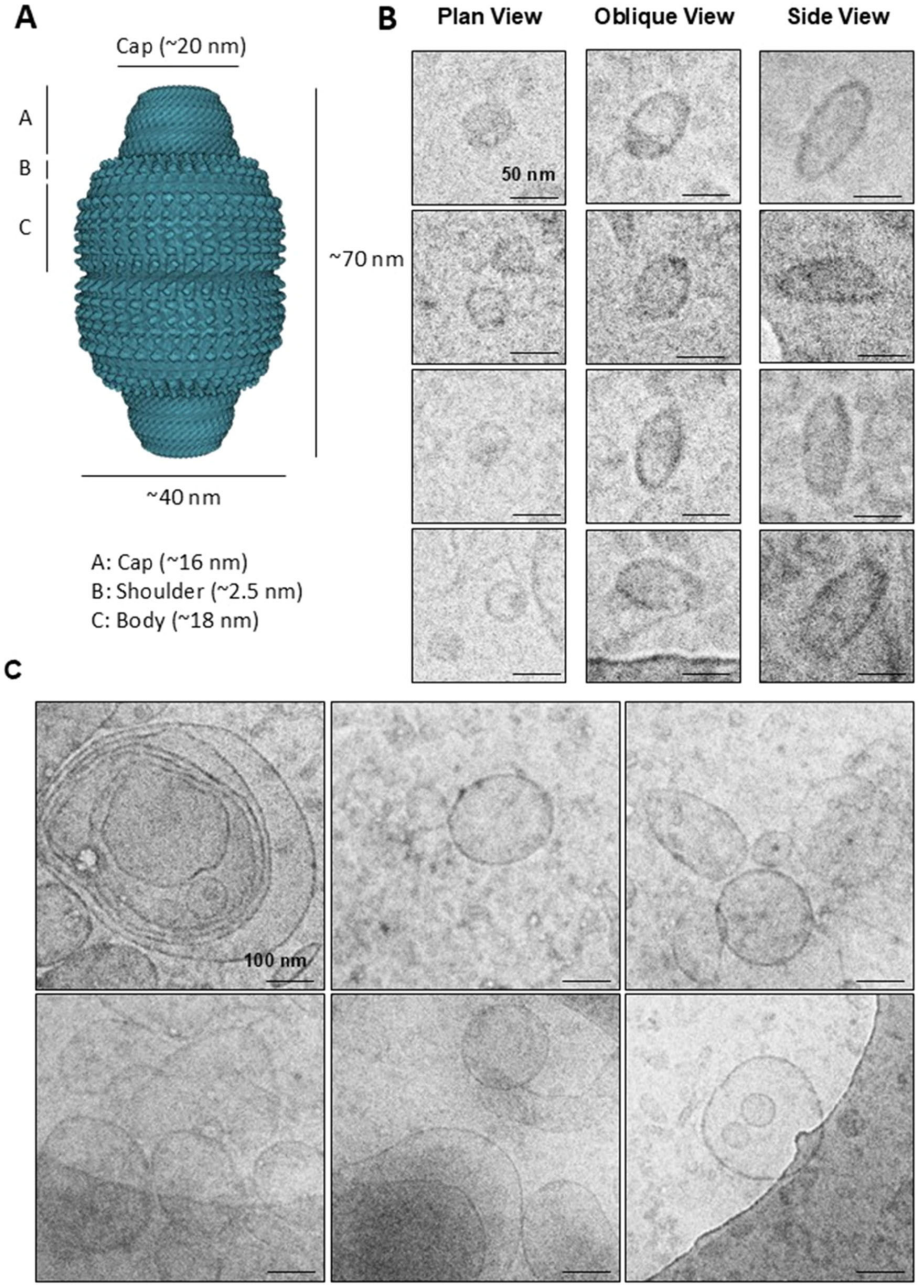
Cryo‐TEM imaging of vault‐like particles and EVs. (A) Structural illustration of vault particle (PDB id: 6BP7). (B) Collage of vault‐like particles in plan (diameter = 41.2 ± 3.8 nm, mean ± SD, *n* = 9), oblique and side view (length = 85.2 ± 9 nm, width = 41.6 ± 3.7 nm, mean ± SD, *n* = 13). Scale bars represent 50 nm. (C) Example images of single and multivesicular EVs ranging from 50 to 500 nm in diameter but not observed to be physically in contact with any vault‐like structure (within expected size range and elliptical shape), on the EV membrane or within EV structures. Scale bars represent 100 nm.

We next examined 100k pellets by cryo‐TEM to determine if intact vault particles were present. We observed barrel‐shaped vault‐like particles measuring 85.2 ± 9 nm × 41.6 ± 3.7 nm (mean ± SD, *n* = 13) (Figure 3A, 3B). We also observed numerous vesicular structures with a clear lipid bilayer in the preparation (Figure 3C). The majority of EVs were single vesicles (100–200 nm diameter) with spherical or oval shapes. There were other rare morphologies, such as triple EVs (i.e. two small EVs contained within a larger EV) and large multilayer EVs (>500 nm diameter). However, vault‐like particles were not found to be physically associated with or within EV structures (Figure 3C and Figure S4).

### 3.2 Vault Particle Proteins Co‐Elute With EVs by Size Exclusion Chromatography

**FIGURE 4:**
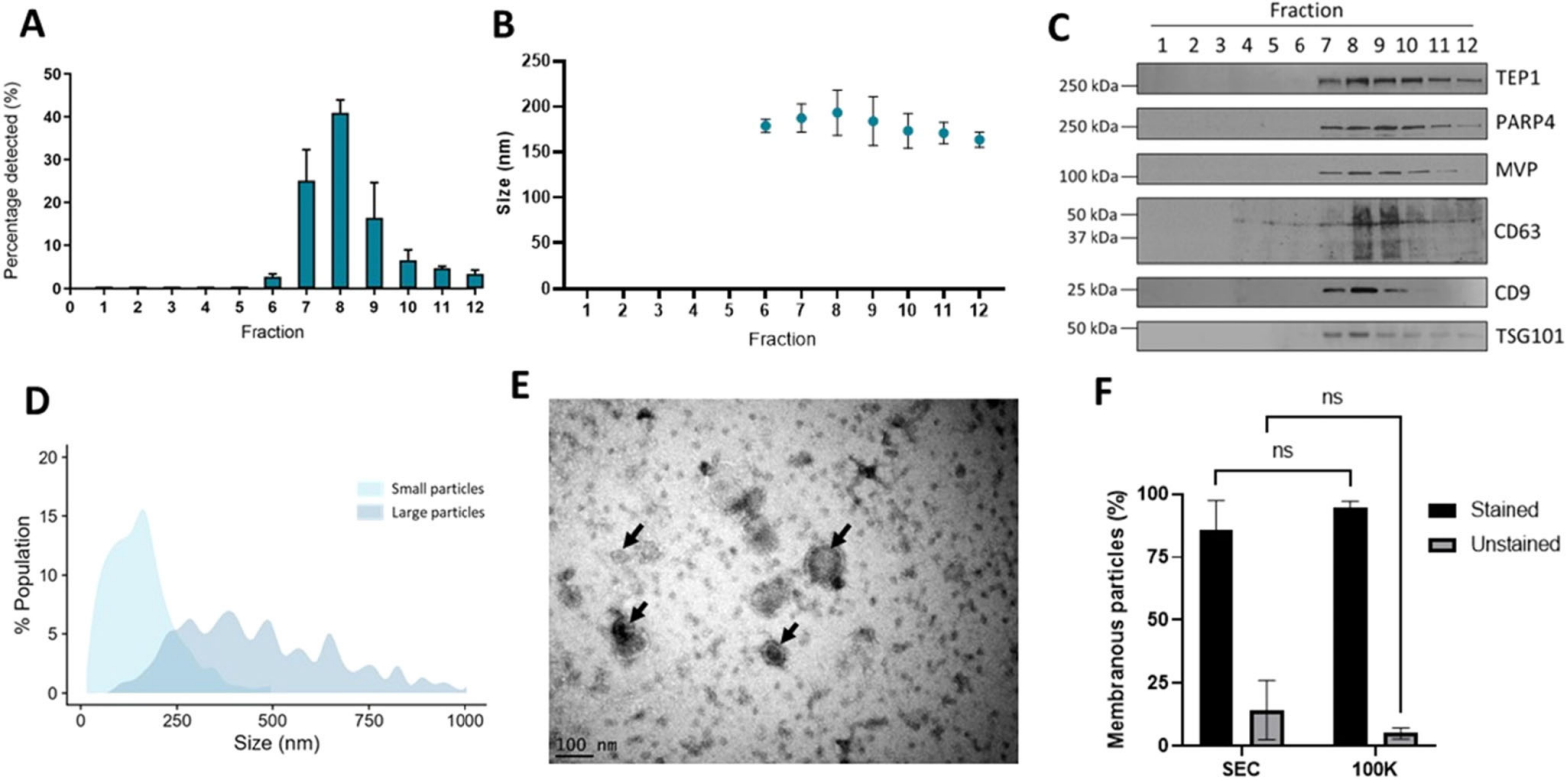
Co‐elution of vault particle and EV markers by size exclusion chromatography. (A) NTA showing SEC particle elution profile (12 × 0.5 mL fractions were collected). Bars represent mean ± SD, *n* = 3. (B) Median particle diameter (nm) present in SEC fractions determined by NTA ± SD, *n* = 3. C) Western blotting to detect vault proteins (TEP1, PARP4, and MVP) and EV markers (CD63, CD9 and TSG101) in SEC fractions. Blots are representative of three independent repeats. (D) SEC fractions 6–11 were combined, particles pelleted by ultracentrifugation at 100,000 *× g* for 1 h, before resuspension in 50 µL PBS and analysed by NTA. Data are mean of three independent experiments. (E) Negative stain TEM analysis of combined SEC fractions. Black arrows indicate individual EVs. (F) Assessment of the proportion of membranous and non‐membranous particles in combined SEC fractions compared to 100k DC pellets. Bars represent mean ± SD, *n* = 3. ns = no statistical difference, two‐way ANOVA.

Having concluded that intact vault particles were present as contaminants in 100k EV pellets, we assessed another well‐established EV isolation technique—size exclusion chromatography (SEC). SCC4 conditioned medium was fractionated by SEC and NTA showed peak particle elution at fraction 8, with the majority of particles being enriched in fractions 7–9 (Figure 4A). The median particle diameter present in SEC fractions was ∼200 nm (Figure 4B). Western blotting of individual SEC fractions revealed that all three vault particle‐associated proteins (TEP1, PARP4 and MVP) co‐eluted with EV makers (CD63, CD9 and TSG101) (Figure 4C). Fractions 6–11 were combined and particles pelleted by ultracentrifugation at 100,000 × *g* for 1 h before resuspension in 50 µL PBS for downstream analysis. NTA was performed using the two different sets of measurement parameters described above. Small particles had a peak diameter of 165 nm with a shoulder peak at 135 nm. Whereas large particles had several prominent peaks between 225–645 nm (Figure 4D). Negatively stained TEM of combined SEC fractions demonstrated a heterogeneous population of cup‐shaped EVs with diameters ranging from 50–100 nm (Figure 4E). We then quantified the proportion of membranous and non‐membranous particles present in the combined SEC fractions by utilising ExoBrite True Membrane staining in conjunction with nano flowcytometry, which revealed that 85.7% of particles were positive for the stain, whereas 14.3% were negative for the stain (Figure 4F). We also attempted to quantify the proportion of membranous and non‐membranous particles in DC‐derived EV pellets. However, 2k and 10k pellets did not contain sufficient particles for the staining protocol. In 100k pellets, 94.9% of particles were positive for the stain and 5.1% of particles were negative for the stain. There was no significant difference in the proportion of membranous and non‐membranous particles in EVs enriched by SEC or in 100k pellets (Figure 4F).

### 3.3 A Vault Particle‐Free EV Isolation Strategy Using Immunocapture

**FIGURE 5:**
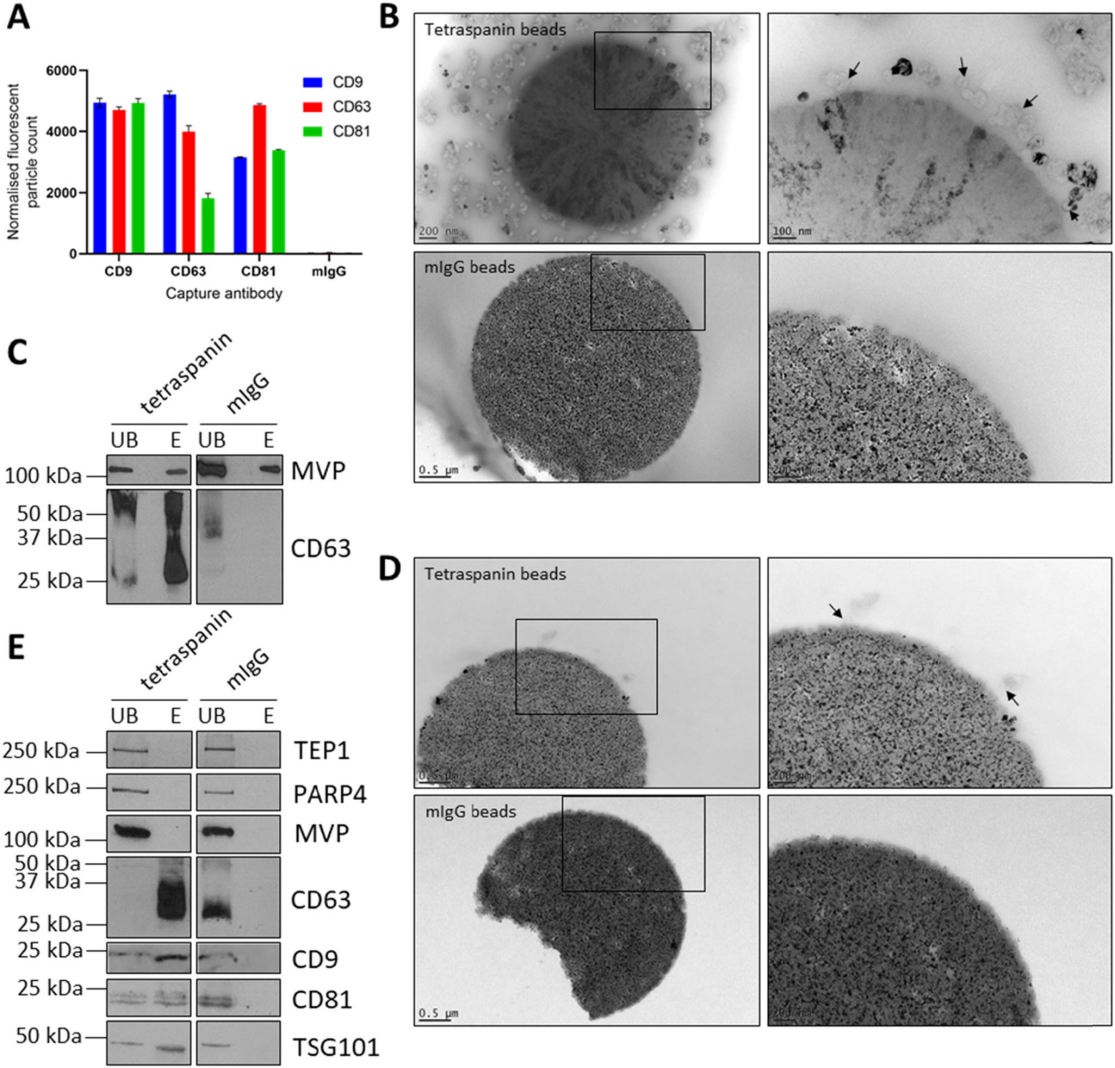
Dynabead immunocapture separates marker‐positive EVs in conditioned medium from vault particles. (A) ExoView analysis of SCC4 cell line conditioned medium using a tetraspanin microchip coated with CD9, CD63, CD81, and mIgG capture antibodies. Captured particles were labelled with fluorescent anti‐CD9, CD63, and CD81 antibodies. Data are means of three technical repeats per capture antibody ± SD. (B) Upper left: CD9/CD63/CD81 Dynabeads capturing differential centrifugation‐derived 100k EV pellets with high level of aggregation. Upper right enlarged view with black arrows indicating EVs; Lower left and lower right: mIgG control Dynabeads with enlarged view. Images were obtained by negatively stained TEM. (C) Western blot detecting MVP and CD63 in unbound and eluted fractions from tetraspanin Dynabeads and mIgG control beads after mixing with resuspended 100k EV pellets overnight. Blots are representative of three biological repeats. (D) Upper left: CD9/CD63/CD81 Dynabeads capturing EVs from conditioned medium with no aggregation. Upper right: enlarged view with black arrows indicating EVs; Lower left and right: mIgG control Dynabeads with enlarged view. Images were obtained by negatively stained TEM. (E) Western blot detecting vault proteins (TEP1, PARP4, and MVP) and EV markers (CD63, CD9, CD81, and TSG101) in unbound and eluted fractions from tetraspanin Dynabeads and mIgG control beads after mixing with concentrated conditioned medium overnight. Blots are representative of three biological repeats.

We progressed to testing commercially available Dynabeads that capture EVs by immunoaffinity, which should reduce contamination with other similar‐sized particles. The above western blotting data and ExoView analysis confirmed that SCC4‐derived EVs were positive for CD9, CD63, and CD81 (Figure 5A). We therefore utilised Dynabeads to capture EVs based on this tetraspanin profile.

To determine whether immunocapture is sufficient to pull out the marker‐positive EVs from DC pellets, we applied the tetraspanin Dynabead purification to resuspend 100k pellets. EV‐Dynabead complexes were examined by TEM, which showed a high level of particle aggregation at the bead surface (Figure 5B), and so this approach was terminated. A preliminary immunoblotting experiment revealed that CD63‐positive EVs and MVP were captured and eluted from the tetraspanin beads (Figure 5C), but confidence was low in this result due to the particle aggregation observed by TEM. We therefore repeated the immunocapture experiment with concentrated conditioned medium that had not been subjected to ultracentrifugation. TEM revealed the capture of individual EVs with minimal aggregation (Figure 5D). To demonstrate that the Dynabead protocol was capturing the targeted EV populations, immunoblotting for all three tetraspanins (CD9, CD63, and CD81) was used, in addition to the luminal EV marker TSG101. Despite the capture of tetraspanin‐positive EVs, all three vault particle proteins (MVP, TEP1, and PARP4) remained in the unbound fraction (Figure 5E).

## 4 Discussion

In recent decades there has been a rapid increase in EV research, with many groups attempting to determine the identity of EV‐associated bioactive molecules and the effect they have upon transfer to recipient cells. Many studies utilise proteomic and transcriptomic approaches to characterise EV protein and RNA profiles, respectively. Vault particle proteins and vtRNAs have been repeatedly reported as EV‐associated molecules or EV cargo (Admyre et al. 2007; Buschow et al. 2010; van Balkom et al. 2015; Xu et al. 2015). More recently, evidence has been presented suggesting that transport of MVP and vtRNAs to the extracellular space is exosome‐independent (Jeppesen et al. 2019).

**FIGURE 6:**
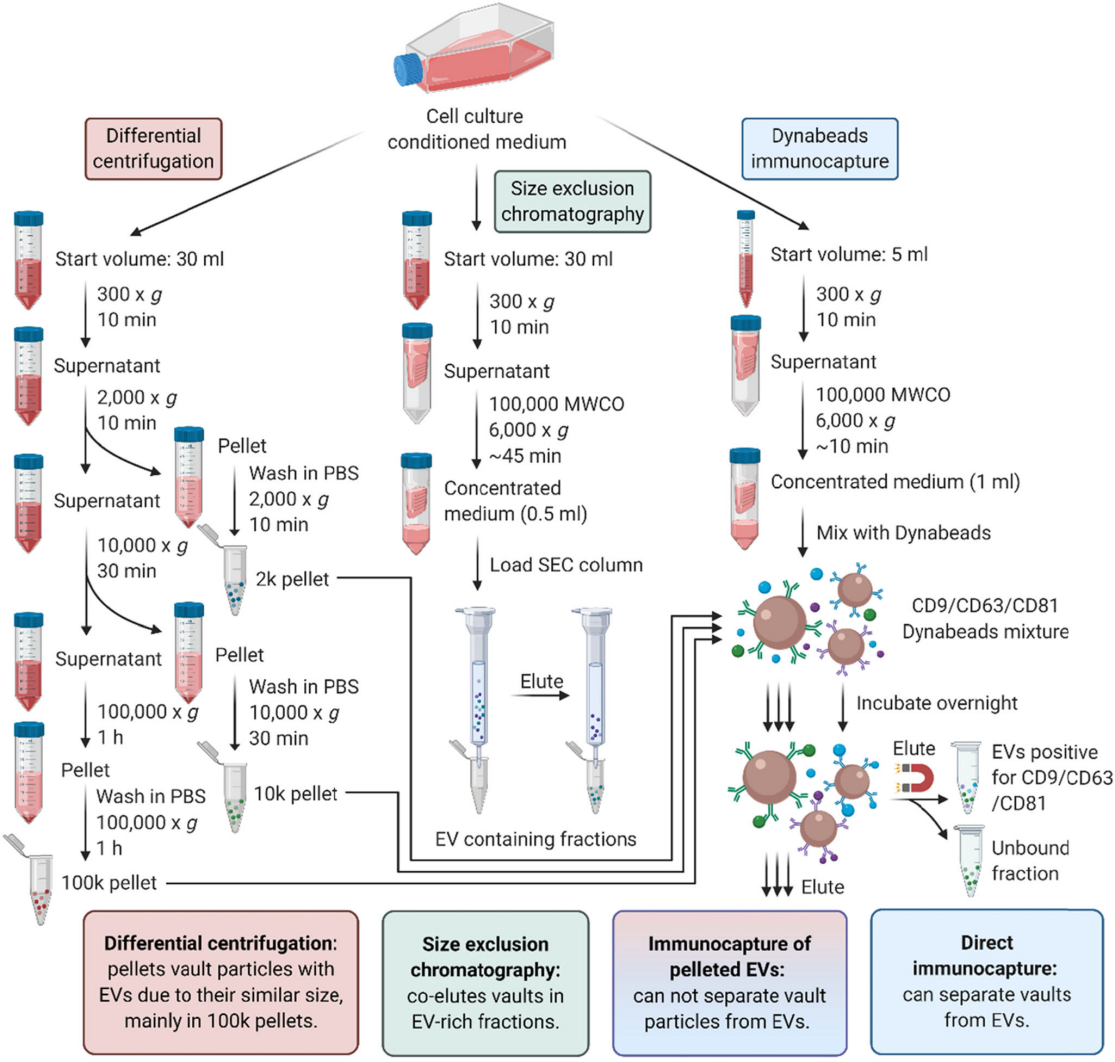
Schematic of EV isolation techniques used in this study and outcomes. EVs from cell culture conditioned medium were isolated by three techniques: differential centrifugation (DC), size exclusion chromatography (SEC), and Dynabead immunocapture. DC and SEC co‐purified vault particles with EVs. Further purification of DC‐derived pellets by immunocapture resulted in particle aggregation and incomplete separation of EVs from vault particles. However, EV capture from concentrated conditioned medium using Dynabeads led selection of marker specific (CD9/CD63/CD81) EVs that were free of vault particle contamination.

To the best of our knowledge, the current study is the first to investigate the topology of vault particle components in EV isolates using biochemical approaches. We determined the association of vault components with EVs isolated by three commonly used techniques (Figure 6). We demonstrated that vault particles are co‐isolated with EVs in 100k pellets from cell culture conditioned medium. Without further investigation, the co‐purified vault particle components would likely be ascribed as EV‐associated molecules. Noticeably, this was identified by the MISEV2018 guidelines as one of the main issues that EV researchers came across, and using biochemical approaches to further demonstrate the topological association of molecules with EVs was highly recommended (Théry et al. 2018).

When viewed from the side, vault particles measure 70 nm in height and 40 nm in width. In plan view (or cross‐section at their widest point), they measure 40 nm in diameter. Hence, they are a similar size to small EVs. They were first discovered as major contaminants of intracellular vesicle preparations when centrifuging whole cell lysates at 100,000 × *g* (Kedersha and Rome 1986). In studies isolating EVs by DC and precipitation techniques, individual vault components have been repeatedly reported as EV cargo. Vault components are often detected in EV preparations, enriching small EVs and exosomes (van Balkom et al. 2015; Shurtleff et al. 2017; Teng et al. 2017). However, we also detected vault components in 2k and 10k DC pellets. This may be due to aggregation and pelleting of smaller particles subject to high centrifugal force.

Proteinase protection assay data showed that MVP, the major structural component of the vault particle, was not protected by an EV membrane in 10k or 100k DC pellets. There was incomplete digestion of MVP in 2k pellets, which we speculate was due to insufficient proteinase concentration or incubation time for the quantity of protein in the 2k pellets. Furthermore, the majority of vtRNAs were degraded when treating 100k pellets with RNase and proteinase (in the absence of detergent), indicating that they are mostly within a protein‐shelled structure like vaults. However, there was incomplete vtRNA degradation, suggesting that some vtRNA may be protected by an EV membrane. It was difficult to draw firm conclusions from the RNAse protection assay data for 2k and 10k pellets due to variation between experimental repeats. These experiments should be repeated but utilising absolute quantification of vtRNA abundance (e.g., digital PCR) to avoid the need for control miRNA for normalisation.

SEC is rapidly becoming one of the most utilised EV enrichment methods. SEC has the advantage of being relatively rapid and yielding more intact, functionally active EVs (Mol et al. 2017; Monguió‐Tortajada et al. 2019), with some studies reporting a particle purity similar to density gradient‐based isolation (Lobb et al. 2015). However, SEC‐derived EVs from human plasma have been shown to be contaminated with albumin and lipoproteins (Baranyai et al. 2015; Stranska et al. 2018). Our data show that vault particle proteins are co‐eluted with EV markers in individual SEC fractions. The presence of all three vault particle proteins (MVP, TEP1 and PARP4) in SEC fractions suggests that intact vault particles are present, but further biochemical experiments are required to test if the vault particle proteins are protected by an EV membrane. In addition, the abundance of vtRNA in SEC preparations should be determined and the RNase protection assay repeated to determine if vtRNA are contained within an EV membrane. The presence of vault particles in SEC preparations should also be confirmed by cryo‐TEM.

Ultracentrifugation has been proposed as a pre‐enrichment method prior to immunocapture (Pedersen et al. 2017). However, our data indicate that high‐speed centrifugation caused particle aggregation, which prevented separation of marker‐positive EVs from MVP. We were able to select tetraspanin‐positive EVs using magnetic bead/antibody complexes and leave behind vault particle proteins when utilising clarified conditioned medium that had not undergone high‐speed centrifugation. However, additional biochemical analysis (RNase and proteinase protection assays) of captured EVs and the unbound fraction is required to determine if vault components are protected by an EV membrane. The presence of vault particles in the unbound fraction should also be confirmed by cryo‐TEM.

We observed vault‐like particles when imaging 100k EV pellets derived from an OSCC cell line by cryo‐TEM. Taken together with the data from Jeppesen et al. (2019), who utilised colon cancer and glioblastoma cell lines, this indicates that this finding is not SCC4 cell line‐specific. Vault components have also been found in EV preparations derived from multiple body fluids and tissues, suggesting that their presence in the extracellular space is not an in vitro cell culture artefact (Admyre et al. 2007; Gonzalez‐Begne et al. 2009; Skogberg et al. 2013; Pienimaeki‐Roemer et al. 2015). It is tempting to speculate that vault export could be the result of a novel mechanism for extracellular secretion of large ribonucleoprotein particles. Nano flowcytometry of lipid‐stained particles present in SEC and 100k DC samples indicated that 14.3% and 5.1% of particles were non‐membranous, respectively. Thus, vault particles are likely to represent a proportion of these particles. However, we are not currently able to quantify the abundance of vault particles present. Although not possible during this study, protein‐to‐lipid ratio measurements (by infra‐red spectroscopy, for example) may be advantageous, but it would be challenging to differentiate between EV‐associated proteins, vault‐associated proteins, or other proteinaceous components. Spectroscopic analysis of pure vault particle preparations may reveal a signature that could be used to determine vault particle abundance in EV preparations. Alternatively, single particle analysis coupled with vault particle tagging could be used in future studies to accurately quantify the proportion of vault particles in EV preparations.

In summary, we have provided compelling evidence that vault particles are present in 100k pellets derived from SCC4‐derived conditioned medium. We also demonstrated the co‐elution of vault particle proteins with EV markers enriched by SEC. We demonstrated that direct immunocapture allows selective enrichment of marker‐positive EVs from as little as 5 mL of conditioned medium. The purified EVs were free of vault particle proteins and can be subjected to downstream analysis. This methodology could also be used as a negative selection strategy to study marker‐negative EV populations or non‐EV extracellular particles like vaults. It would also be beneficial to develop vault particle isolation strategies, either by immunocapture (potentially using antibodies against MVP) or density gradient ultracentrifugation. This would allow the functional study of vault particles that had been separated from EVs and other nanoparticles.

## Author Contributions

**Xinming Liu**: Data curation (lead), formal analysis (lead), investigation (lead), methodology (lead), visualization (lead), writing – original draft (lead), writing – review and editing (equal). **Zubair A Nizamudeen**: Data curation (supporting), formal analysis (supporting), investigation (supporting), methodology (supporting), visualization (supporting), writing – original draft (supporting), writing – review and editing (equal). **Christopher Hill**: Investigation (supporting), methodology (supporting), resources (supporting), writing – original draft (supporting), writing – review and editing (equal). **Christopher Parmenter**: Investigation (supporting), methodology (supporting), resources (supporting), writing – original draft (supporting), writing – review and editing (equal). **Kenton P Arkill**: investigation (supporting), methodology (supporting), resources (supporting), visualization (supporting), writing – original draft (supporting), writing – review and editing (equal). **Daniel lambert**: conceptualization (equal), funding acquisition (supporting), project administration (supporting), supervision (supporting), writing – original draft (supporting), writing – review and editing (equal). **Stuart Hunt**: Conceptualization (equal), data curation (supporting), formal analysis (supporting), funding acquisition (lead), methodology (supporting), project administration (lead), resources (lead), supervision (lead), visualization (supporting), writing – original draft (supporting), writing – review and editing (equal).

## Conflicts of Interest

The authors declare no conflicts of interest.

## Supporting information




**Supporting Figures and Tables**: jev270142‐sup‐0001‐SuppMat.docx

## Data Availability

Small RNA sequencing data is available for download via the University of Sheffield data repository (ORDA) (https://doi.org/10.15131/shef.data.25152620.v1). All other data that support the findings of this study are available from the corresponding author upon reasonable request.
