# Supplementary material for: Assessment of Vault Particles in Cancer Cell Line‐Derived Extracellular Vesicle Preparations
Source: J Extracell Vesicles. 2025 Aug 6;14(8):e70142. doi: 10.1002/jev2.70142 (PMC12326185; doi:10.1002/jev2.70142)
Supplement: Supplementary file 1 — Supporting Figures and Tables: jev270142‐sup‐0001‐SuppMat.docx [file JEV2-14-e70142-s001.docx]

**Supplementary data**

**Table S1. Summary of small RNA sequencing for the EV pellets from H357 and SCC4.**


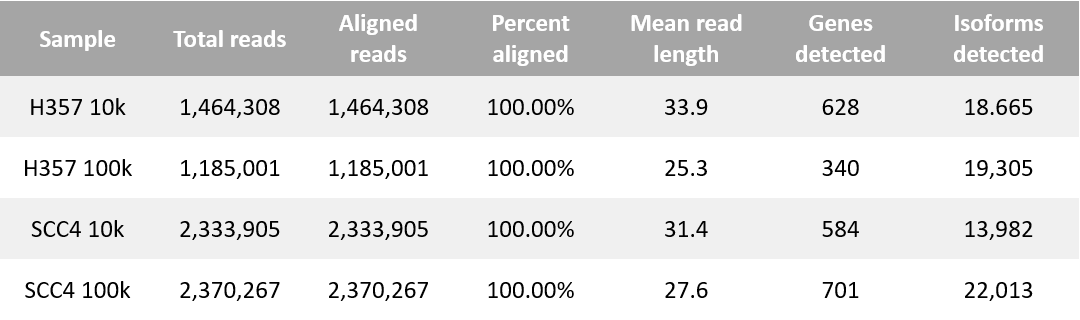


**Table S2. A selection of miRNA reads in small RNA-seq data from H357 and SCC4 derived DC pellets.**


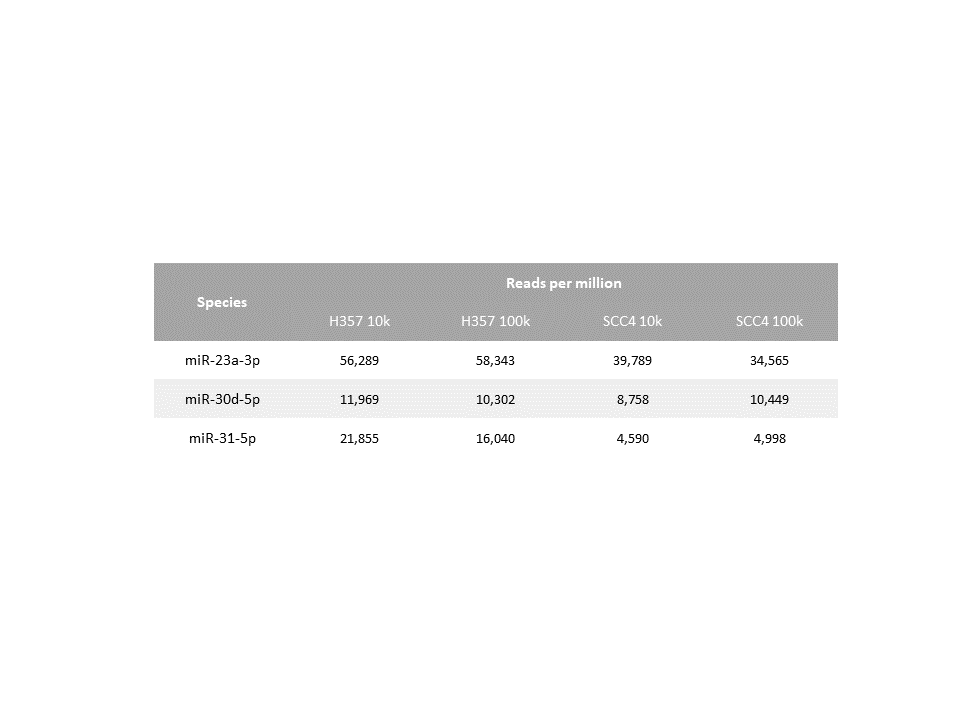


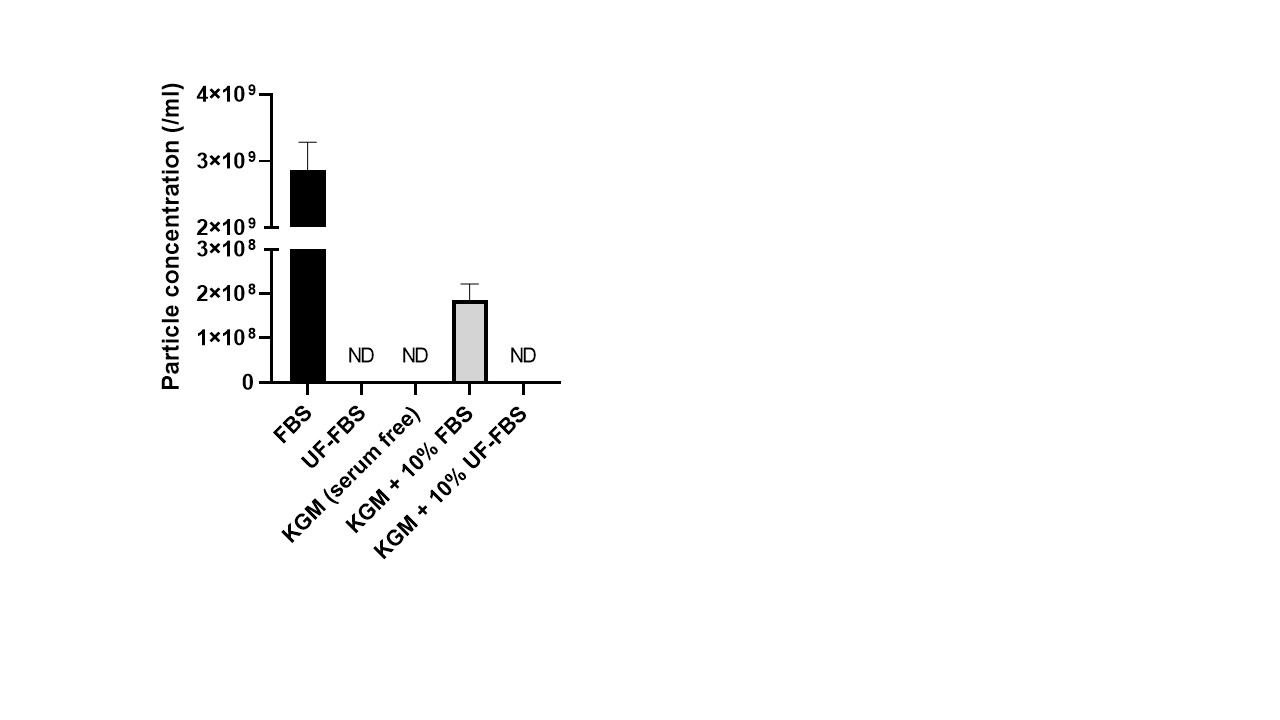


**Figure S1. Analysis of particle concentration in UF-FBS.**

UF-FBS was prepared according to the method of Kornilov et al. (2018). The particle concentration of FBS, UF-FBS, serum-free KGM and KGM supplemented with either 10% (v/v) FBS or 10% (v/v) UF-FBS was determined by NTA. Data are means ± SD, n=3, ND = not detected.


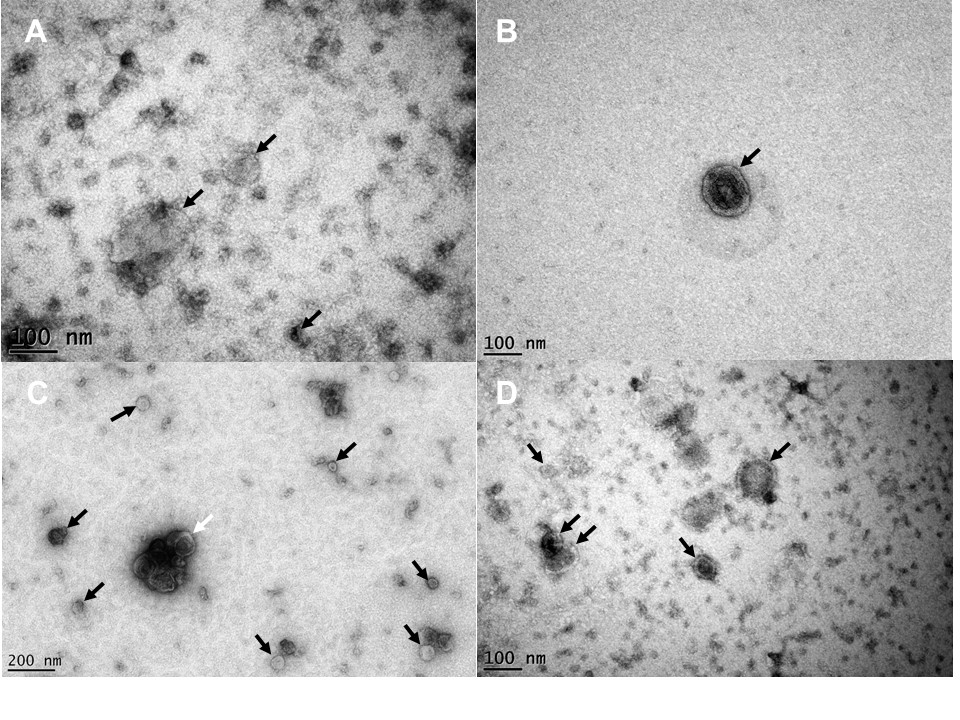


**Figure S2. Enlarged negatively stained transmission electron micrographs.**

Negative stain TEM analysis of **(A)** 2k pellet, **(B)** 10k pellet, **(C)**100k pellet and **(D)** SEC enriched EVs from SCC4 cell line. Black arrows indicate individual EVs, and the white arrow indicates an aggregate of EVs.


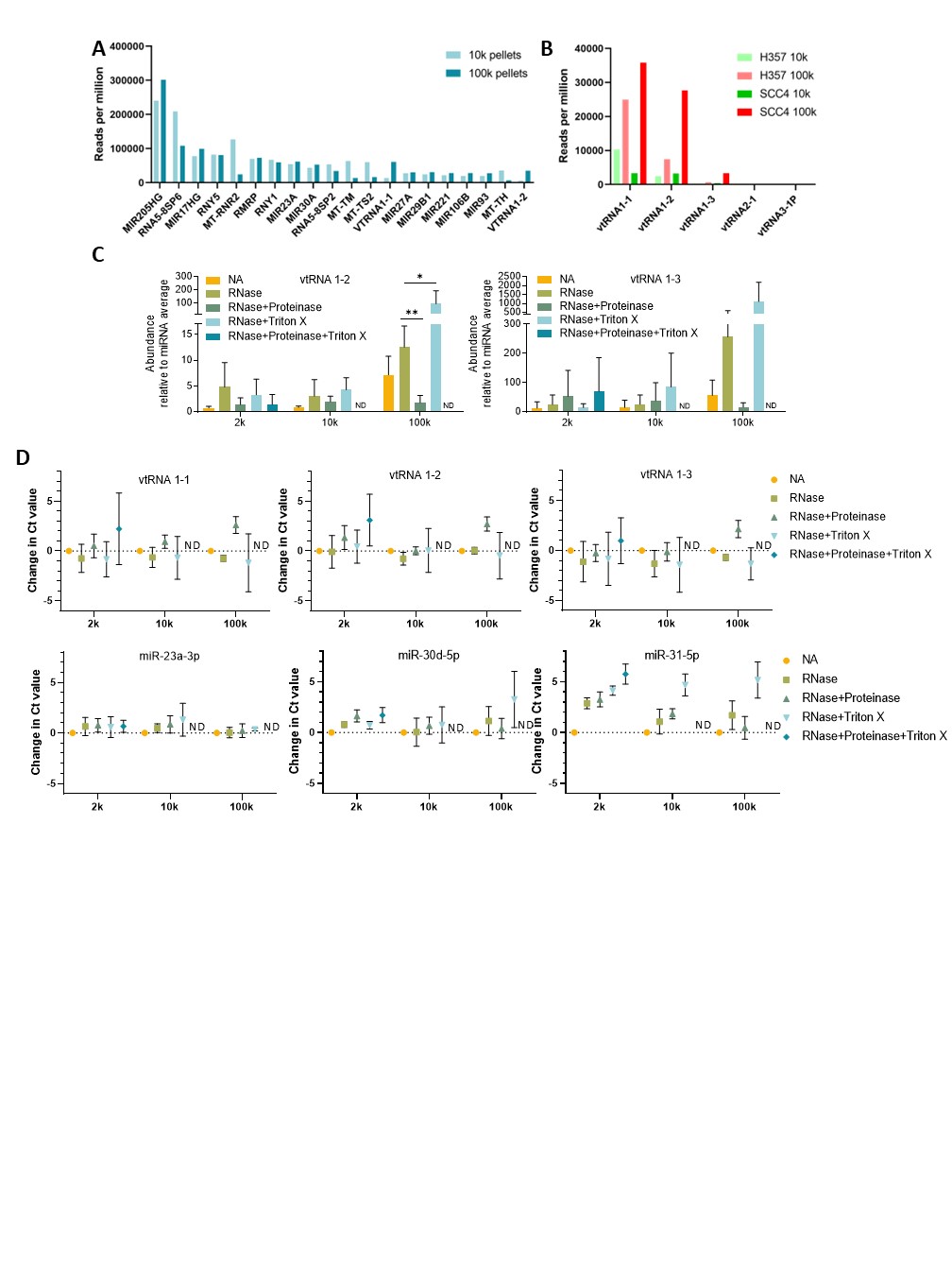


**Figure S3. Summary of small RNA sequencing and RNase protection assay.**

**A)** The top 20 most enriched small RNA species in H357 and SCC4-derived DC pellets, ranked by total reads in all samples. **B)** vtRNA reads in H357 and SCC4 DC pellets determined by small RNA sequencing **C)** RNase protection assay followed by qPCR shows vtRNA1-2 and vtRNA1-3 abundance upon DC pellet treatment. Data are means ± SD, n=3, ND = not detected due to insufficient RNA material for qPCR analysis after treatment. Statistical significance was assessed by multiple t tests corrected with the Holm-Sidak method, **p*<0.05, ***p*<0.001. **D)** Change in Ct value for vtRNA 1-1, vtRNA 1-2, vtRNA 1-3, miR-23a-3p, miR-30d-5p, and miR-31-5p following RNase protection assay, data were normalised to the NA group. Data are means ± SD, n=3, ND = not detected due to insufficient RNA material for qPCR analysis after treatment.


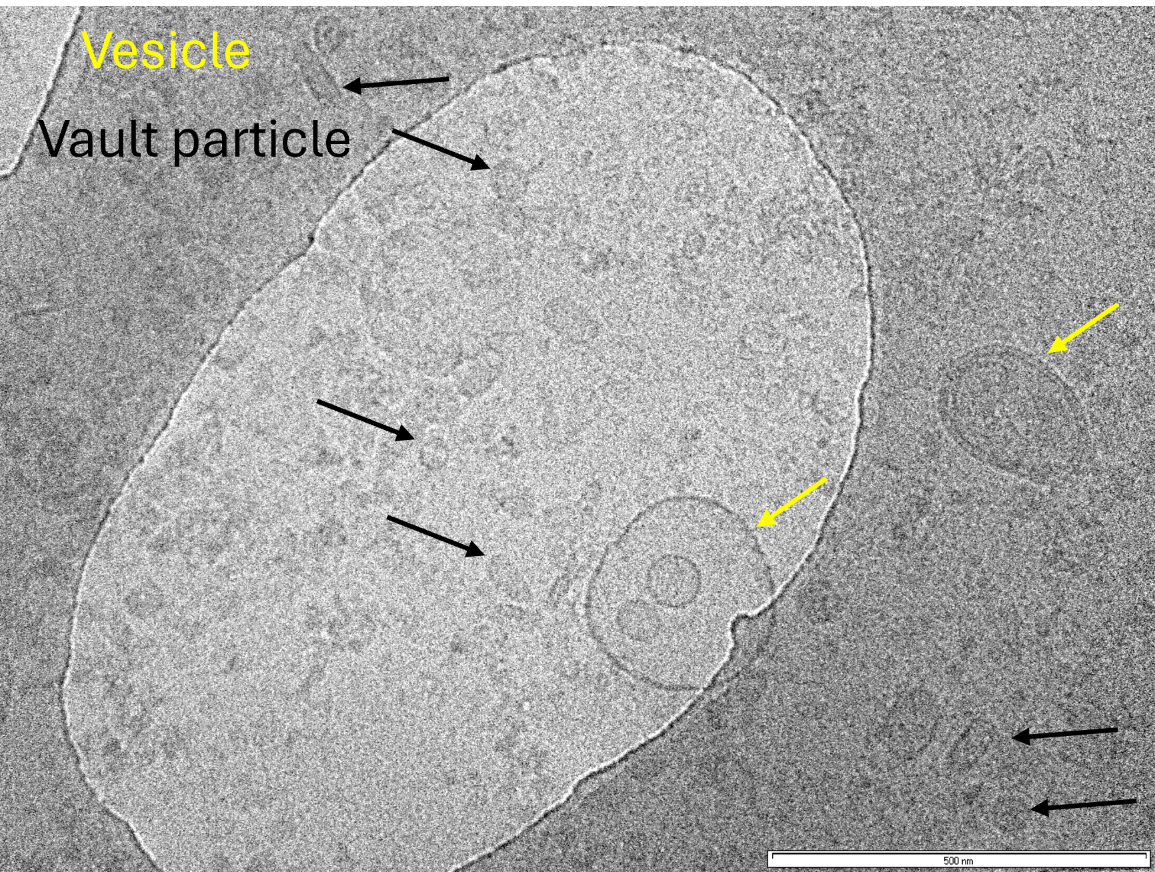


**Figure S4. Uncropped Cryo-transmission electron micrograph.** EVs are indicated by yellow arrows and vault-like particles are indicated by black arrows.
